# Laboratory set-up for OBEs and projections

**DOI:** 10.3389/fpsyg.2026.1879392

**Published:** 2026-10-01

**Authors:** Giovanni B. Caputo

**Affiliations:** Department of Humanistic Studies (DISTUM), University of Urbino, Urbino, Italy

**Keywords:** absorption, Cotard’s syndrome, dissociative identity, materiality, meditation, mindfulness, skin, spirituality

## Abstract

Eye-to-eye gazing under low-level illumination provides a controlled laboratory paradigm for investigating out-of-body experiences (OBEs) and psychological projections. In the present study, a large sample of healthy participants (*N* = 398) completed a dyadic gazing task. Principal Component Analysis (PCA) conducted on nine items of the Strange-Face Questionnaire (SFQ) yielded a three-factor solution: (i) facial visual deformations (Derealization / Detachment-of-Reality; DR factor); (ii) out-of-body experiences (OBE factor); and (iii) apparitional projections of “ancestors” (Projection factor). Structural Equation Modeling (SEM) and Path Analysis were employed to test the relationships among these constructs. Results demonstrated that OBEs play a pivotal role in facilitating the projection of “ancestors” onto the partner’s face, revealing a direct predictive path from disembodiment (OBE) to projective phenomena. This pathway generalized to other apparitional percepts, including the projection of a “child,” “adolescent,” or “monster.” Path Analyses further indicated that external reality serves a broader function than merely triggering perceptual distortions; rather, it operates as a “gate” regulating the inhibition versus excitation of internal projections depending on whether the projective content is “positive” (ancestor, adolescent, child) or “negative” (monster). We hypothesize that interoceptive/proprioceptive embodied simulation–driven by non-conscious, premotor mimicry of the partner’s facial expressions and respiratory rhythm–provides the somatic scaffolding required to bind elements from memory into multimodal consciousness. This mechanism allows the mind to dynamically shift processing between exteroceptive facial input, autobiographical memory, inherited facial engrams, and archetypal facial images. Finally, this research bridges experimental psychology with analytical psychology by mapping the projected figures of ancestor, adolescent, child, and monster onto Jung’s core archetypes: the Wise Master (*Philemon/Baucis*), Perennial Youth (*Anima/Animus*), the Divine Child (*Puer Aeternus*), and the Shadow, respectively. “Positive” versus “negative” archetypes were operationalized using Path Analysis. Finally, Network Analysis maps the systemic interconnectedness among all seven archetypal projections identified across the SFQ items–which, in addition to the four core archetypes, include *Hermaphroditus*, *Animal*, and *Lapis*.

## Introduction

Strange-face illusions are fundamentally face-specific phenomena, directly reflecting the neural specialization of cortical structures dedicated to face processing. These illusions can be reliably induced by instructing individuals to maintain steady fixation on a face under low-level illumination for a prolonged duration. The paradigm operates effectively whether viewing one’s own reflection in a mirror (Caputo, 2010) or observing another person’s face in a dyadic setup (Caputo, 2019; see Figure 1).

**FIGURE 1:**
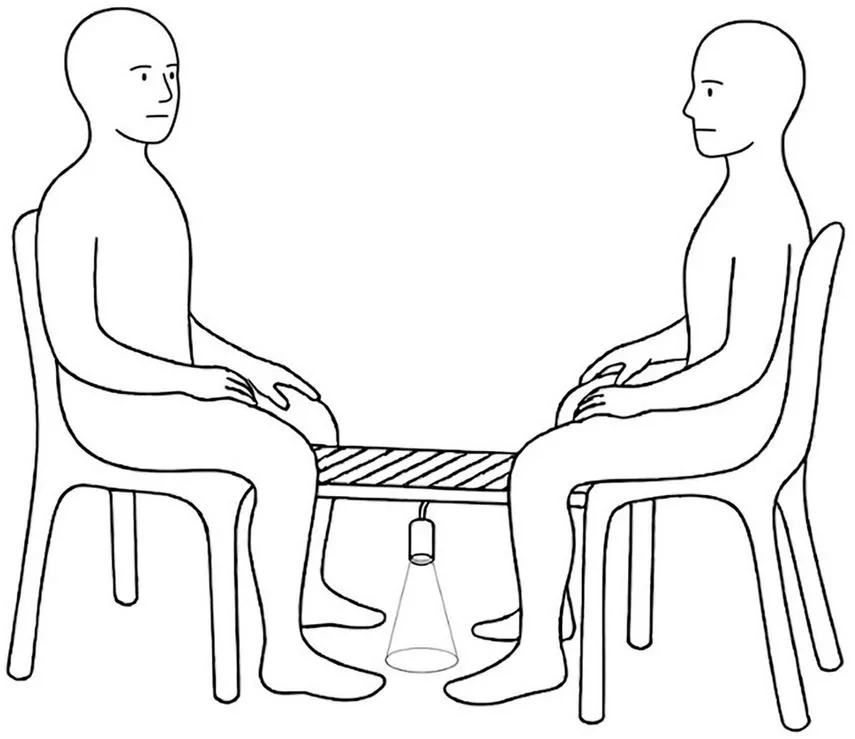
The experimental sessions were conducted in a controlled environment consisting of a dark room approximately 3 m × 3 m in size. The sole illumination source was a spotlight mounted beneath an opaque partition situated between the two subjects. To ensure indirect, ambient lighting, the spotlight was directed toward the floor. The spotlight must point exactly to the room’s geometric center to avoid lighting asymmetries and artifacts. The resulting illuminance at the facial level was maintained at approximately 0.8 lux, creating the necessary low-light conditions for the induction of strange-face illusions. The opaque panel mounted between the two chairs is intended to prevent the participants’ faces from being illuminated from below. Subjects were seated such that the inter-head distance was approximately 1 m, facilitating direct eye-to-eye contact throughout the 10-min duration of the trial. Participants should maintain a relaxed, neutral facial expression and look directly into each other’s eyes. Modified from Caputo (2019).

Low-level physiological mechanisms alone cannot adequately account for strange-face illusions. For example, neither the Troxler effect (the perceptual fading of isoluminant peripheral stimuli during sustained fixation; Troxler, 1804) nor the Brewster illusion (peripheral object degradation under similar conditions; Brewster, 1818) explains the sudden, apparitional emergence of entirely new faces directly at the central point of fixation. Instead, closer parallels exist in specific neuropsychological conditions where face hallucinations present as complex visual events driven by hyperactivation or misfiring within the brain’s face-processing circuitry–most notably the fusiform face area (FFA) within the ventral occipitotemporal cortex. In Charles Bonnet syndrome, severe sensory deprivation reduces visual input, triggering deafferentation-driven visual release phenomena wherein the visual cortex spontaneously generates vivid images of faces and people, all while the patient retains full insight into their hallucinatory nature (Christoph et al., 2025). Similarly, visual hallucinations in Parkinson’s disease often begin as subtle misperceptions or an heightened susceptibility to visual pareidolia before progressing to fully formed illusions of human or animal faces (Göbel et al., 2021).

To psychometrically capture these experiences, specific categories of illusions–including facial deformations, bodily detachment, and face misidentifications–were initially hypothesized *a priori* (Caputo, 2023) and subsequently validated empirically using the Strange-Face Questionnaire (SFQ), an instrument specifically developed for this paradigm. In a recent manuscript, Caputo and Daniels (2026) performed a Principal Component Analysis (PCA) on the 33 items of the SFQ in a large non-clinical sample, extracting three core factors. Subsequent hierarchical regression analyses using the three subscales of the gold-standard Dissociative Experiences Scale (DES; Carlson and Putnam, 1993; Dubester and Braun, 1995) as predictors revealed that these SFQ factors specifically index three dissociative constructs: derealization, depersonalization, and dissociative identity.

The SFQ derealization subscale originally comprised seven items capturing perceptual phenomena ranging from deformations of facial features to temporal distortion and loss of agency. The SFQ depersonalization subscale similarly contained seven items spanning out-of-body experiences, body-schema distortions, and facial gestalt misperceptions. In the present study, these derealization and depersonalization subscales were each refined to three highly coherent items to establish a distinct, psychometrically robust separation between these constructs. Specifically: (1) Factor 1 (Derealization/Facial Deformations, DR): Captures both localized feature distortions and holistic facial deformations, isolated using SFQ items 1, 29, and 32; (2) Factor 2 (Depersonalization/Out-of-Body Experiences): Operationalizes OBEs using SFQ items 17, 20, and 22.

Figure 2 illustrates an artist’s rendition capturing these joint DR and OBE experiences during dyadic gazing. Actually, this portrait was created by the artist, who was naive about psychological constructs and theories, during sessions other than the experimental sessions reported here.

**FIGURE 2:**
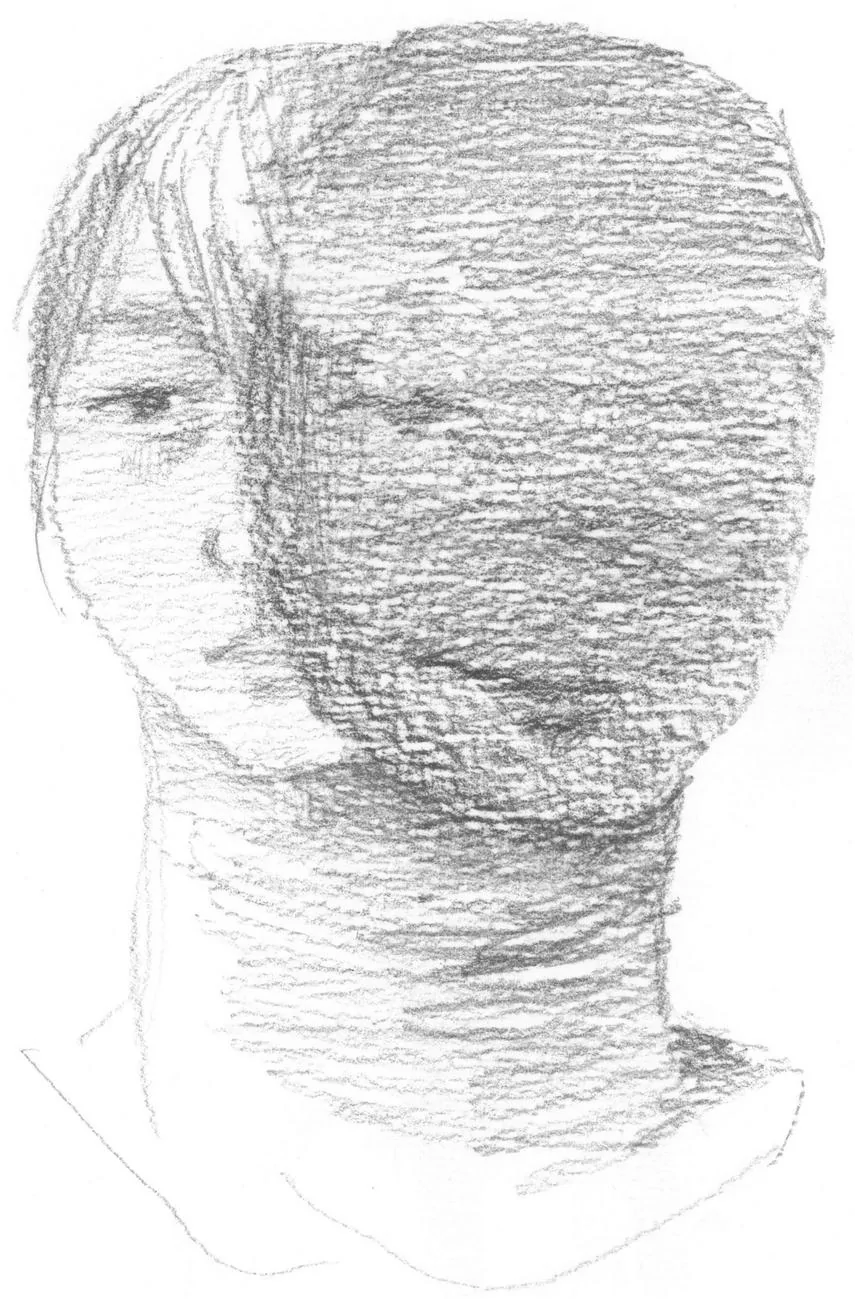
This drawing illustrates both the “deformation” (i.e., derealization, DR) and “detachment” (i.e., out-of-body experience, OBE) of the other’s face as experienced by a portrait artist. In reality, this pristine drawing was captured during a separate eye-to-eye gazing session, distinct from the main sessions discussed here. Because the artist was entirely naïve to strange-face illusions, the portrait perfectly exemplifies how the various psychological factors involved in these illusions naturally overlap. Presumably, the nude skeleton of the face precedes the binding of a prototype.

Crucially, the DES dissociative amnesia subscale uniquely and specifically predicted the third SFQ factor. In the original 33-item PCA by Caputo and Daniels (2026), this third factor exhibits the highest inter-individual variability among the extracted components, encompassing the recognition of both familiar individuals and “strange beings.” Over time, it has received various theoretical designations, including “face misidentification” (Caputo and Daniels, 2026), “dissociative identity” (Caputo, 2023), and “alienating identity” via “disembodiment from the image” (Pennisi, 2025). The present article resolves the ambiguity of this third factor by demonstrating its intrinsic polymorphism and multifaceted nature, which comprises multiple, partially independent identity prototypes.

Understanding this third factor requires grounding in the clinical literature on dissociative identity disorder (DID; formerly multiple personality disorder). Diagnostic guidelines characterize DID by the presence of two or more distinct personality states accompanied by profound dissociative amnesia (American Psychiatric Association, 2013). These recurrent, altered consciousness states routinely govern behavior, with each state potentially possessing its own name, age, gender, personal history, and distinct physical traits. Crucially, this dissociative amnesia manifests as recurrent gaps in daily recall directly tied to internal multiplicity–meaning identity states typically lack mutual awareness, leaving one identity unable to recall the actions or memories of another (Dorahy, 2022; Taïb et al., 2023). Building on these clinical traits, we posit that a similar multiplicity of identity states can be experimentally induced and operationalized within the controlled laboratory set-up (Figure 1) designed to elicit face-specific dissociative states in healthy individuals.

Given the broad phenomenological variability observed in this third stage–where participants perceive diverse identities such as ancestors, adolescents, children, or monsters (Caputo, 2010)–we propose “Projection” as the most theoretically fitting descriptor for this third factor. Within psychological literature, projection is defined as “the process by which one attributes one’s own individual positive or negative characteristics, affects, and impulses to another person or group” (APA Dictionary of Psychology). Freud (1911/1958) originally introduced projection in the context of paranoia, postulating that “an internal perception is suppressed, and, instead, its content, after undergoing a certain kind of distortion, enters consciousness in the form of an external perception” (p. 66). Jung (1969) significantly expanded this scope, arguing that projection is not restricted to “negative” traits–the externalization of disowned, undesirable impulses–but also encompasses “positive” projections involving unacknowledged potential, latent creativity, or idealizations. Jung (1979) noted that “projections change the world into the replica of one’s own unknown face” (para. 19), viewing them as fundamental mechanisms of individuation–the psychological integration of disparate psychic elements toward realizing the Self (Jung, 2009).

Integrating these frameworks, we hypothesize that the various identities perceived in the other’s face during dyadic gazing represent projective phenomena displaying rich phenomenological diversity. The objective of the present study was to identify and categorize these distinct projective typologies within a large sample of young adults while statistically controlling for derealization (DR) and depersonalization (OBE) factors. Specifically, we examined the apparitional projection of *Ancestors*, alongside related developmental projections such as an *Adolescent* or a *Child*. Conversely, we evaluated “negative” apparitional experiences–specifically the perception of *Monsters*–to determine whether they differ fundamentally from positive projections by exhibiting a higher propensity for abrupt perceptual emergence from the dyadic partner’s face. Finally, these results were extended to other projections identified across the SFQ items, including *Hermaphroditus* (i.e., the apparitional projection of an androgyne), *Animal* (i.e., the apparitional projection of a domestic or wild animal), and *Lapis* (i.e., the apparitional projection of an inanimate face)–projected in the partner’s face.

## Materials and methods

### Sample-size power analysis

Sample size determination followed the standard psychometric guideline recommending a minimum ratio of 10 participants per item for questionnaire validation. For the 33-item Strange-Face Questionnaire (SFQ), this requirement indicated a minimum target of 330 participants. For Structural Equation Modeling (SEM) and Path Analysis, Kline (1998) recommends a case-to-parameter ratio of at least 10:1 (and ideally 20:1), with an absolute lower bound of 200 cases. The achieved sample size (*N* = 398) satisfies both criteria, providing adequate statistical power for oblique factor rotations, path models, and network estimations.

### Participants

A total of 414 first-year undergraduate psychology students participated in the study in exchange for course credit. The study was advertised with the following description: *“The lab session entails staring into another person’s eyes for 10 min. In addition, you will be asked to complete several questionnaires regarding potentially distressing past and present experiences.”* Demographic data collected during informed consent indicated a mean age of 19.92 years (*SD* = 2.14, range: 17–36). The initial sample comprised 302 females, 102 males, and 10 non-binary individuals.

All participants were naive to the experimental hypotheses and unfamiliar with strange-face illusions. None had previously participated in experimental sessions involving mirror-gazing or dyadic eye-to-eye gazing procedures.

Data screening followed a systematic exclusion cascade. Three participants were unable to complete the 10-minute eye-to-eye gazing task (*n*_nonresponders_ = 3), requiring the exclusion of their dyadic partners (*n* = 3), resulting in 408 participants who successfully completed the protocol. There were no “omitters” (*n*_omitters_ = 0), i.e., no participant responded compulsively “No, never” across all SFQ items. Subsequently, 10 participants (*n*_biasers_ = 10) were excluded due to affirmative endorsement of the control item (item 19) of SFQ, indicating an acquiescent response bias. Following these screening procedures, the final sample consisted of *N* = 398 individuals. Across this final sample, the mean item endorsement frequency was 49.42%.

### Measures

The Strange-Face Questionnaire (SFQ; Caputo, 2023) was administered following the 10-min dyadic gazing session to assess perceptual and dissociative illusions. The SFQ is a self-report instrument scored on a 5-point scale ranging from “No, never” to “Very often,” with intermediate anchors for “Rarely,” “Sometimes,” and “Often.” Thirty-three items measure specific phenomenological experiences involving perceptual facial distortions (e.g., item 1: “Did you see that some facial features were deformed?”) and altered bodily self-location (e.g., item 22: “Did you perceive that you were looking at the face from outside the body?”).

To adapt the instrument from a mirror setup to a dyadic context, item 15 from Caputo’s (2023) original mirror-gazing scale (“Did you get the feeling of being looked at through the mirror by another person?”) was replaced with item 15 from Caputo (2019): “Did you see that the face of the other had something in common with your face?” The SFQ demonstrated good internal consistency in the present sample (Cronbach’s *alpha* = 0.88), comparable to previous validations under eye-fixation controlled mirror-gazing conditions (*alpha* = 0.83; Caputo, 2023).

### Procedure

Participants were randomly assigned to dyads prior to testing and entered the laboratory in pairs. Participants of the dyad were seated on chairs facing each other (Figure 1), their heads approximately 1 m apart replicating the lab set-up of a previous study (Caputo, 2019). After switching the lights to 0.8 lux of face illuminance, participants were asked to maintain uninterrupted eye contact with each other for 10 min. Participants were instructed as follows: *“Please maintain a neutral facial expression. Keep gazing continuously into the eyes of the other participant. The session will last ten min.”* Following the gazing task, participants completed the SFQ digitally via Qualtrics using forced-response settings, eliminating missing item-level data.

### Statistical analysis

Principal Component Analyses (PCAs) were conducted to evaluate item clustering and factor retention for the Projection dimensions. To accommodate expected inter-factor correlations, PCAs were executed using Promax (oblique) rotation with factor extraction set to eigenvalues greater than 1.0. For reporting clarity, factor loadings below 0.30 were suppressed in Tables.

The Detachment-of-Reality (DR) factor was anchored by items 1, 29, and 32, while the Out-of-Body Experience (OBE) factor was anchored by items 17, 20, and 22. Four distinct Projection models were tested, each centered on a primary prototypical item: *Ancestor* (item 26), *Adolescent* (item 9), *Child* (item 7), and *Monster* (item 4). Other items were selected via PCAs among items that were loading into the same Projection factor with the prototypical item.

Path Analyses were conducted using Structural Equation Modeling (SEM) with Robust Maximum Likelihood (MLM) estimation to account for minor multivariate non-normality. Model fit was evaluated using the Comparative Fit Index (CFI), Tucker-Lewis Index (TLI), and Root Mean Square Error of Approximation (RMSEA). The primary structural path evaluated across models specified that Projection is predicted by Detachment-of-Reality (DR) and Out-of-Body Experiences (OBE).

Finally, a Gaussian Graphical Model (GGM) Network Analysis was estimated across all extracted Projection items using the Extended Bayesian Information Criterion and graphical LASSO regularization (EBICglasso).

## Results

Preliminary analyses revealed no statistically significant differences in SFQ item responses as a function of participant gender (*p* > 0.05) or age (*p* > 0.30). Factor structures and Path Analysis for the *Ancestor* projection onto the dyadic partner’s face are presented in Figures 3A,B, respectively. Corresponding models for the *Adolescent* and *Child* projections are illustrated in Figures 4A,B, 5A,B. Across all three positive projection models, global fit indices indicated excellent model fit (CFI ≈ 1.00, TLI ≈ 1.00, RMSEA < 0.001).

**FIGURE 3:**
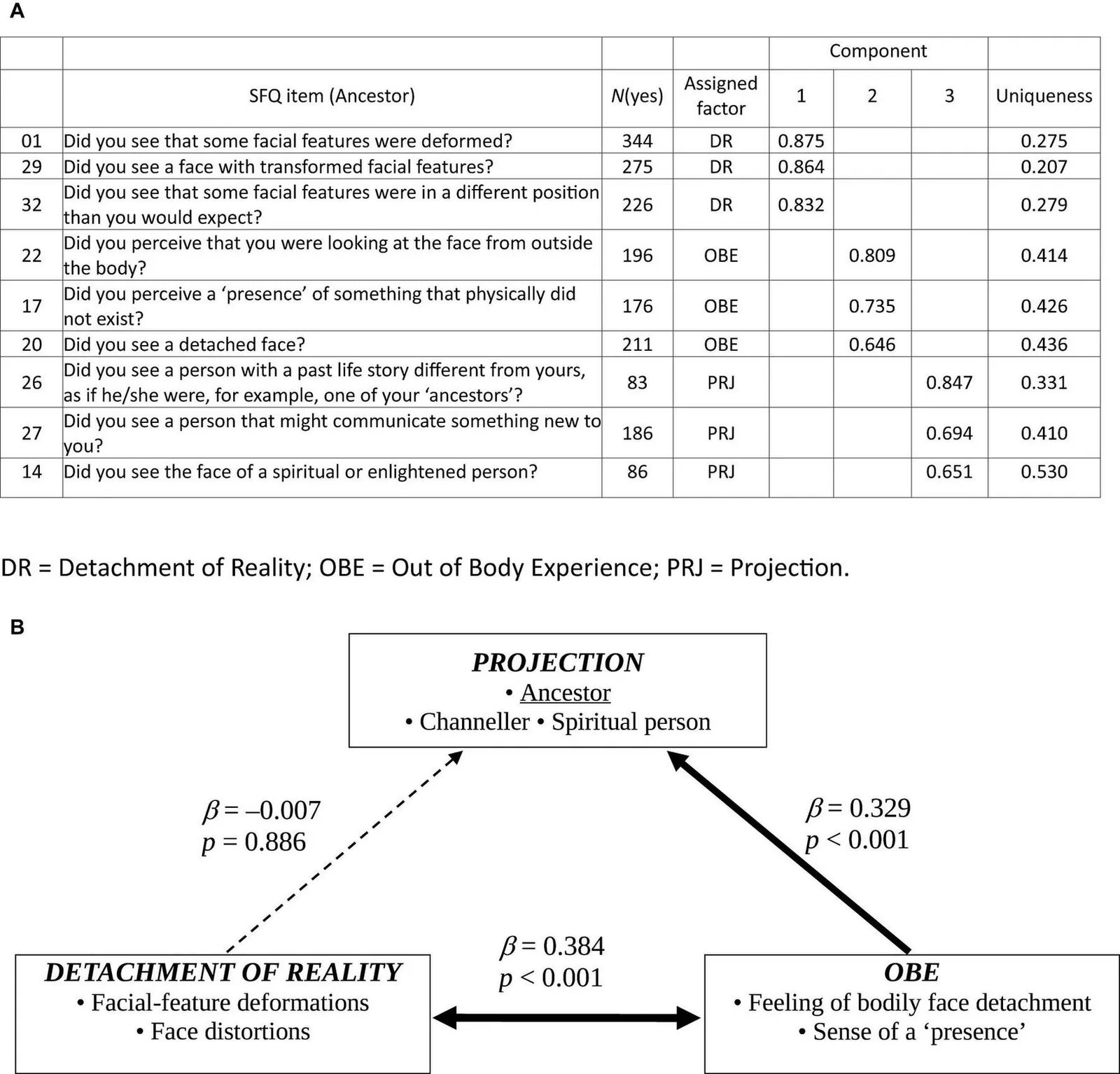
**(A)** A factor analysis yielded two fixed factors (DR and OBE) and a third factor (Projection). This third factor centered on the apparitional experience of an “Ancestor” (item 26) projected onto the face of the other participant in the dyad. This prototype clustered with two other items, characterizing the ancestor as a “spiritual” entity (item 14) attempting to communicate something to the subject (item 27) through the mediation of the other dyad member (i.e., the “channeller”). Principal Component Analysis (PCA) was performed, with factor extraction based on eigenvalues greater than 1.0. To account for potential correlations between factors, a Promax (oblique) rotation was applied. For clarity, factor loadings with coefficients lower than 0.3 were suppressed and omitted from the final matrix. The resulting model accounted for 63% of the total explained variance. The variable *N*(yes) represents the number of participants who endorsed a specific item with an affirmative response, out of a total sample size of *N* = 398. **(B)**. Path Analysis for the “Ancestor” prototype. Path Analysis involves tripartite factors in low-illumination eye-to-eye gazing (*N* = 398). This study utilizes path analysis to examine the relationships between three primary factors emergent during dyadic eye-to-eye gazing under low-level illumination: (i) Exteroceptive Processes: Perceived deformations and distortions of facial features (DR) are driven by exteroceptive sensory data derived from the other’s face. (ii) Interoceptive/Proprioceptive Processes: Conversely, out-of-body experiences (OBEs) and the “sense of a presence” are rooted in interoceptive and proprioceptive bodily information. These states likely manifest through the non-conscious mimicry of the other’s facial expressions and emotional states. (iii) Embodied Projections: Apparitional projections of alternative personalities–such as latent dissociative identities of ancestors, “channeled” entities, or spiritual figures–become “embodied” within the other’s face. The model indicates that a detachment of external reality (DR) is significantly correlated with OBEs, with a primary link leading from OBEs to apparitional projections. Furthermore, an inhibitory link (dashed line) exists between exteroception (DR) and projection. While this link did not reach statistical significance in the current sample, it likely functions as a “gate” mechanism that regulates projections and facilitates a “reset” to external reality. During the onset of apparitional visions, this inhibitory gate may be suppressed. This suppression results in a disinhibition (or excitation) of the pathway, allowing distorted facial features to trigger apparitional projections via the mediating influence of OBEs.

**FIGURE 4:**
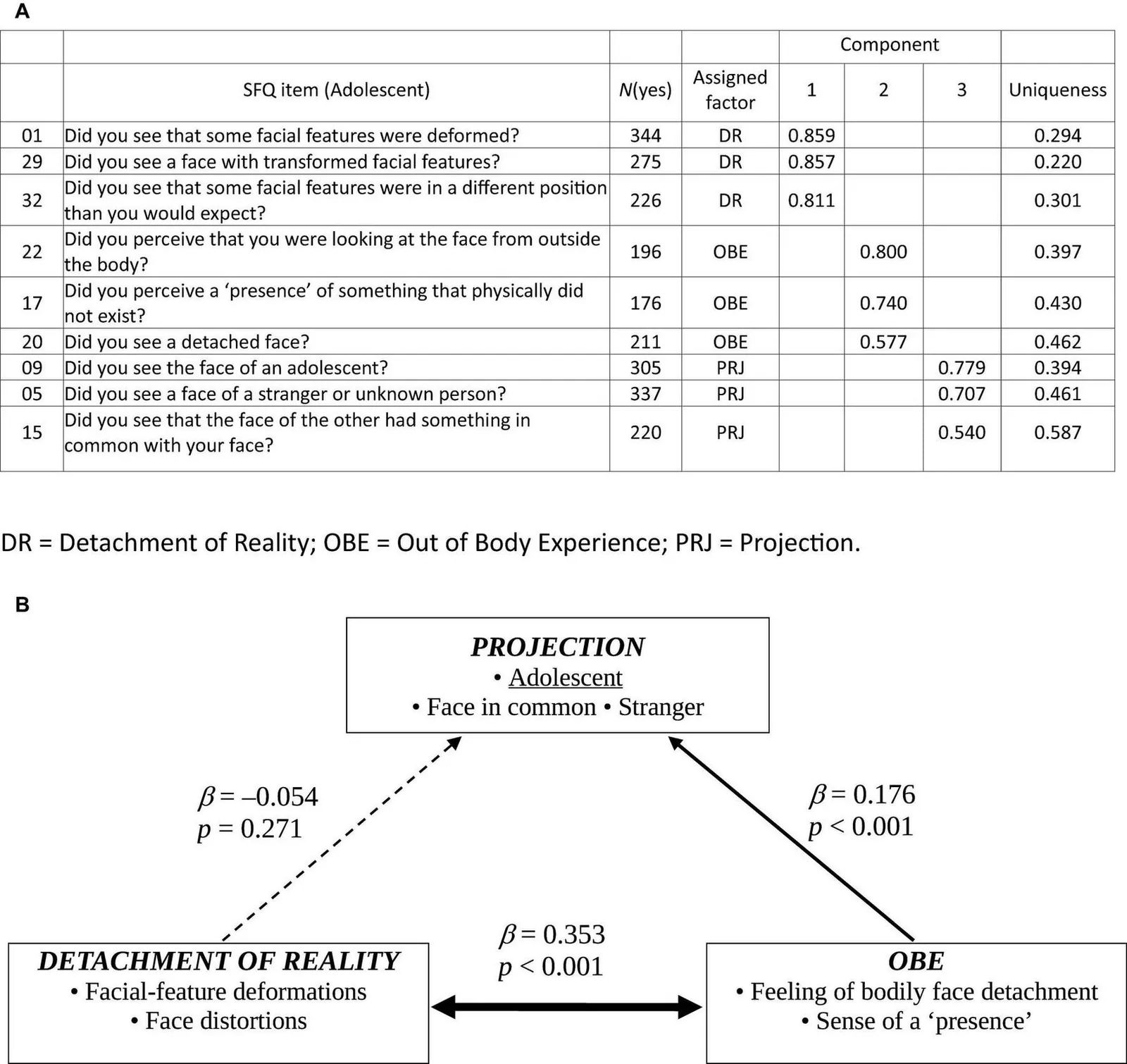
**(A)** Results of a Principal Component Analysis. A factor analysis yielded two fixed factors (DR and OBE) and a third factor (Projection). This third factor centered on the apparitional experience of an “Adolescent” (item 9) projected onto the face of the other participant in the dyad. This prototype clustered with two other items, characterizing the adolescent as a “stranger” entity (item 5) inviting a fusional experience (item 15) with the subject. The resulting model accounted for 61% of the total explained variance. **(B)** Path Analysis for the “Adolescent”prototype. Apparitional projections of alternative personalities–such as latent dissociative identities of an adolescent, “face in common” entities, or strange figures–become “embodied” within the other’s face.

**FIGURE 5:**
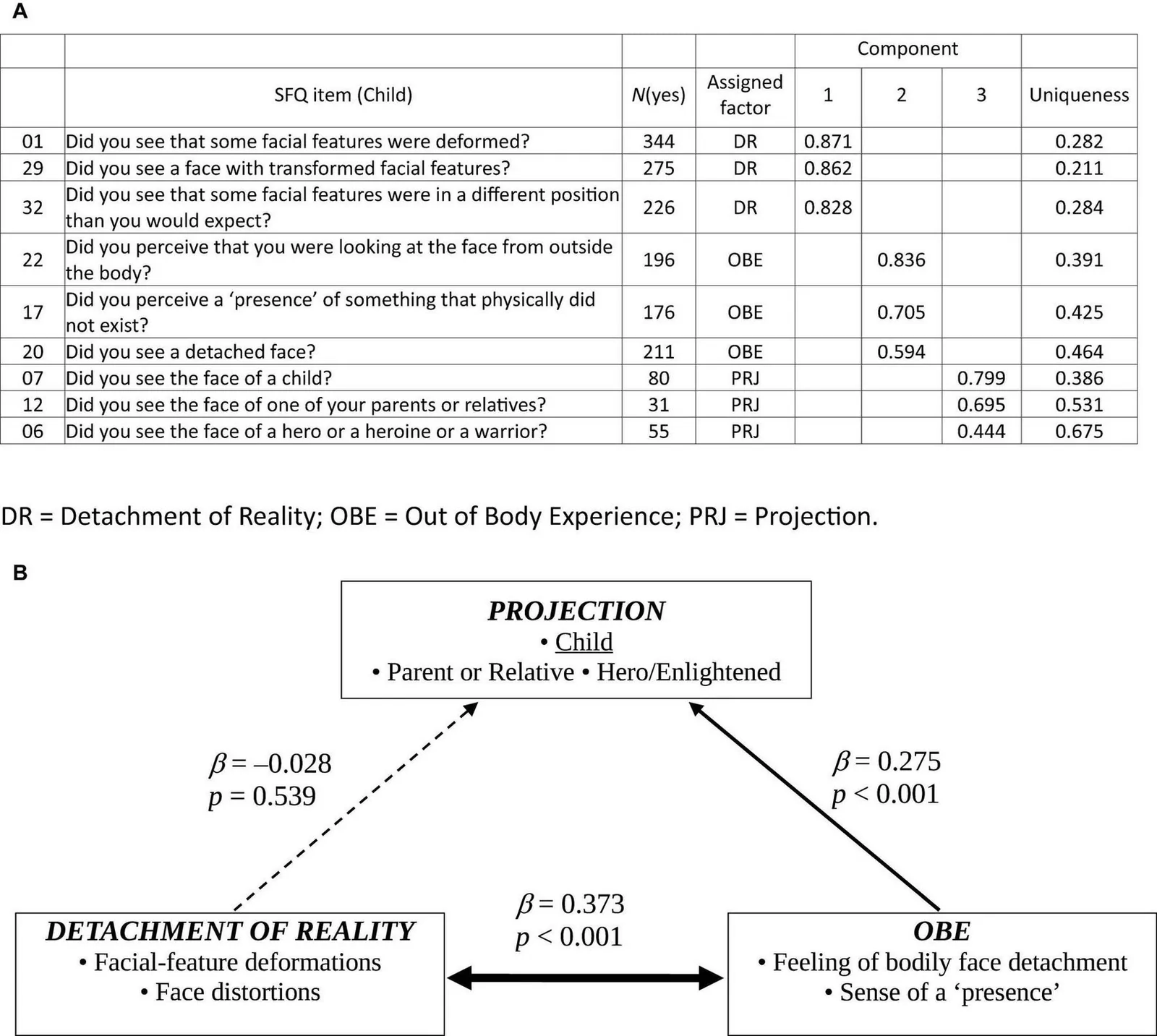
**(A)** Results of a Principal Component Analysis. A factor analysis yielded two fixed factors (DR and OBE) and a third factor (Projection). This third factor centered on the apparitional experience of a “Child” (item 7) projected onto the face of the other participant in the dyad. This prototype clustered with two other items, characterizing the child as a “parent or relative” entity (item 12) acting as a hero/heroine or a warrior (item 6). The resulting model accounted for 60% of the total explained variance. **(B)** Path Analysis for the “Child” prototype. Apparitional projections of alternative personalities–such as latent dissociative identities of a child, a parent or relative, or an enlightened hero–become “embodied” within the other’s face in the dyad.

The traits of spirituality (item 14) and heroism (item 6) cross-loaded onto both the *Ancestor* and *Child* projection factors. To maintain construct purity, these items were assigned to the factor on which they exhibited the higher primary loading.

Projections of an *Old Person* (item 8) were empirically distinct from projections of an *Ancestor* (item 26) or a *Spiritual Entity* (item 14). Rather than reflecting venerated lineage, the *Old Person* projection captured somatic aspects of physical aging–such as deeply wrinkled skin, swollen eyelids, altered lip contours, and depressed expressions. Consequently, item 8 clustered directly with the *Monster* prototype rather than the positive *Ancestor* prototype.

The *Adolescent* prototype (item 9) formed a factor characterized by a dialectical tension between opposing states: perceived identity fusion with the partner’s face (item 15) co-occurring with feelings of strangeness against them (item 5). This dialectic of opposites encapsulates the vital energy of the Adolescent prototype, which fundamentally parallels the immediate “here-and-now” experience of adolescence (Jensen and Nutt, 2016).

An overarching PCA conducted across these three positive projection clusters confirmed a three-factor solution (Figure 6), demonstrating structural independence among the *Ancestor*, *Adolescent*, and *Child* prototypes despite the shared variance between Ancestor and Child figures on spiritual/heroic dimensions.

**FIGURE 6:**
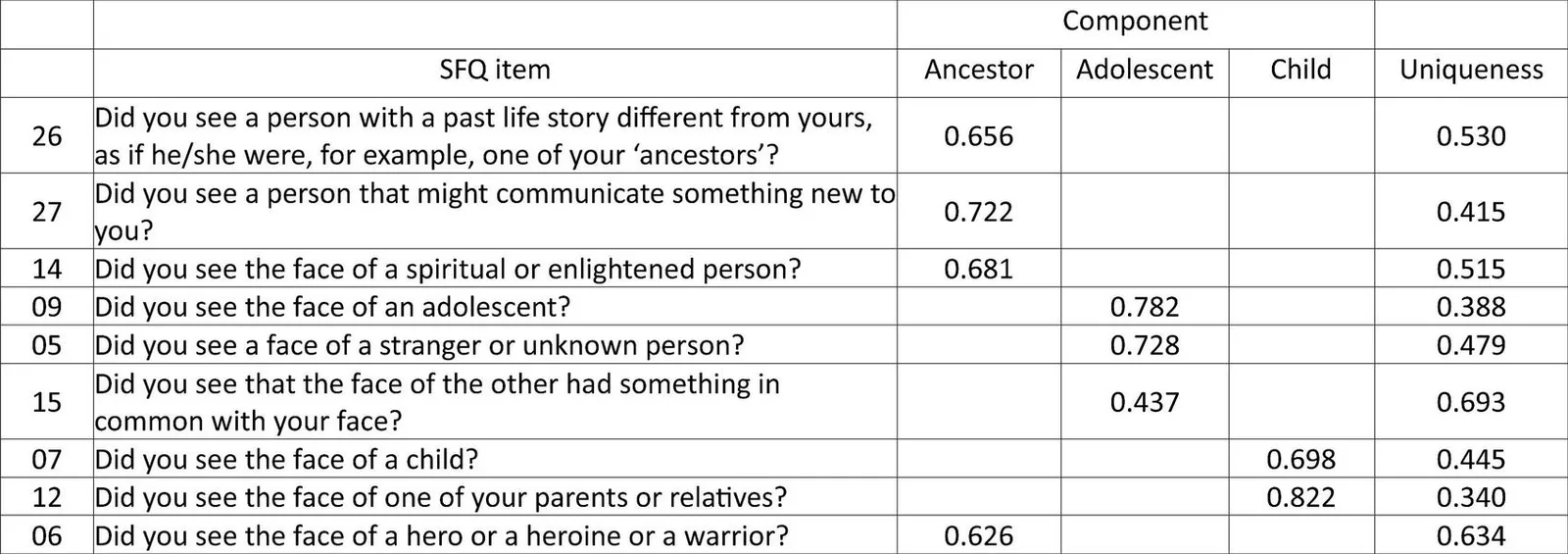
Principal Component Analysis (PCA) of the three “positive” prototype projections–the Ancestor, the Adolescent, and the Child–as they are projected onto the face of the other member of the dyad. Explained variance was 51%.

Across Figures 3B, 4B, 5B, the path models demonstrate a consistent pattern for positive projections: out-of-body states (OBE) exert a direct, positive effect on Projection, whereas derealization (DR) displays an inhibitory (negative) path to Projection.

In contrast, the projection of a *Monster* (item 4; Figure 7A) revealed a fundamentally different regulatory architecture (Figure 7B). The *Monster* prototype clustered with *Old Person* (item 8) and *Alien* (item 21). Path Analysis (Figure 7B) demonstrated an excitatory (positive) path linking derealization (DR) directly to the Projection of monstrous figures, establishing a distinct statistical pattern that characterizes “negative” projections.

**FIGURE 7:**
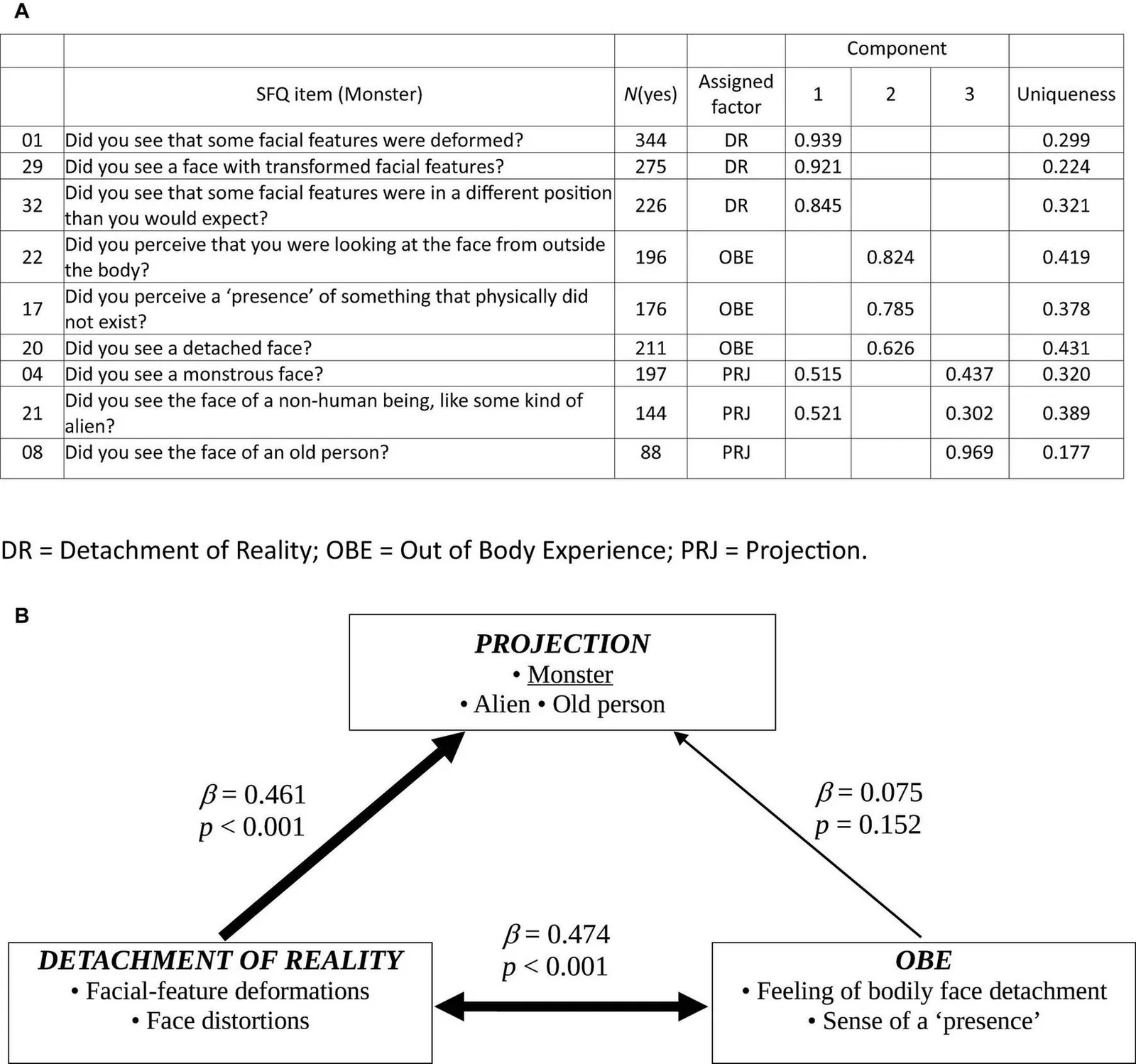
**(A)** Results of a Principal Component Analysis. A factor analysis yielded two fixed factors (DR and OBE) and a third factor (Projection). This third factor centered on the apparitional experience of a “Monster” (item 4) projected onto the face of the other participant in the dyad. This prototype clustered with two other items, characterizing the monster as an “alien” entity (item 21) or an “old” person (item 8). The resulting model accounted for 67% of the total explained variance. It is important to note that two items associated with the Projection factor also exhibited significant cross-loadings on the Detachment-of-Reality factor. This secondary loading suggests a conceptual overlap between these two constructs, indicating that these specific items may capture elements of both dissociative derealization and the externalization of internal projections. **(B)** Path Analysis of the “Monster” prototype. Within the dyadic gazing paradigm, apparitional projections of alternative personalities–including latent dissociative identities characterized as monsters, aliens, or old figures–become “embodied” upon the other’s face. This phenomenon represents a cognitive-perceptual shift where internal memories or dissociative fragments are transposed onto external sensory stimuli. Statistical modeling reveals a direct excitatory link (continuous line) between Detachment-of-Reality and the projection of monstrous identities. This specific pathway suggests two critical interpretations: (1) Projective Facilitation: Monstrous prototypes may possess a lower threshold for activation, making them significantly easier to project onto the other’s facial features than other personality constructs; (2) Perceptual Delusion: These monstrous projections may be experienced with a heightened sense of “subjective reality.” Consequently, the subject may perceive these distorted images as being indistinguishable from–or even more authentic than–objective external reality.

## Discussion

Under conditions of low-level illumination, the laboratory paradigm of dyadic eye-to-eye gazing reliably induces face-specific illusions. The reduction of ambient visual input decreases exteroceptive perceptual processing, thereby increasing the relative salience of interoceptive and proprioceptive signals. Consequently, these somatically driven feelings play a critical role in bypassing the normal visual-cognitive constraints of facial recognition. Bodily states such as felt facial detachment and OBEs systematically unsettle standard visual-cognitive processing, generating complex strange-face illusions projected onto the partner’s actual face.

The primary empirical finding of this study highlights the pivotal role of OBEs in facilitating the projection of strange-face illusions. This mechanism appears to operate via an initial phase of somatic disembodiment, during which the participant’s connection to their own embodied facial schema is temporarily suspended. Once this somatic anchor is disrupted, the partner’s face functions as a neutral substrate onto which subjective mental representations–sourced either from internal memories or latent prototypical facial structures–can be transposed. In this context, pre-projective OBEs serve as the necessary somatosensory scaffolding or integrative matrix required to anchor a projected visual identity onto the partner’s physical features.

This model diverges fundamentally from traditional motor simulation theories of empathy, originally proposed by Lipps (1909) and expanded by Gallese (2025) following the discovery of mirror neurons. While motor simulation models prioritize motoric mirroring, our model demonstrates that the core integrative mechanism in these illusions relies on the interoceptive and proprioceptive representation of the bodily self. This conceptualization aligns closely with Metzinger’s (2009) framework regarding the aggregative dominance of the bodily self. According to Metzinger, the internal representation of one’s own body serves as the primary structural framework for binding multimodal exteroceptive input and interoceptive signals. When this self-body model is experimentally destabilized–as observed in the present paradigm–it opens a processing window for the multimodal integration of projected internal representations onto the partner’s bodily self within the subject’s mind.

Throughout this process, the perceptual phenomenon remains localized within the subject’s mind, while the partner in the dyad facilitates the illusion by serving as an interactive “mirror” via non-conscious facial mimicry (Hess and Fischer, 2022). The underlying binding mechanism is generated by somatic detachment; this state of depersonalization renders the partner’s “disembodied” facial substrate highly receptive to internal mental content. While these bound representations can theoretically incorporate features from the immediate environment, our experimental configuration deliberately minimizes objective visual cues. By relaxing these external constraints, the protocol prioritizes the emergence of internal, memory-based representations. These projections may draw from autobiographical memory–such as the likeness of a parent or relative–or manifest as unfamiliar, strange entities when prototypical face schemas abruptly supersede the partner’s actual features.

The influence of the external environment is most pronounced at the onset of the projective process, manifesting as localized feature distortions and holistic deformations of the facial Gestalt. These distortions correlate strongly with OBEs, suggesting a reciprocally modulating relationship wherein visual degradation and somatic disembodiment mutually reinforce one another. However, Path Analysis reveals a critical divergence in how these dissociative states dictate specific projective outcomes. While OBEs directly facilitate the apparitional projection of structured entities–such as an *Ancestor*, *Adolescent*, or *Child*–a distinct inhibitory path emerges between derealization (DR) and projection. This inhibitory pathway points to a homeostatic regulatory mechanism–a “perceptual reset”–that promotes realignment with objective sensory input, thereby suppressing active projections and constraining visual distortions to preserve baseline cognitive stability.

Crucially, this regulatory reset mechanism fails to operate during the apparitional perception of *Monsters*. In contrast to “positive” projections, a strong excitatory path links derealization (DR) directly to the projection of monstrous entities. These findings demonstrate a marked perceptual bias toward negative projections; specifically, “negative” monstrous apparitions are projected onto the partner’s face with greater ease than “positive” figures. Furthermore, their association with derealization suggests that monstrous projections carry a heightened degree of delusional conviction, leading participants to experience these threatening entities as more real or “objective” than the partner’s actual face.

Methodologically, the eye-to-eye paradigm–alongside mirror-gazing configurations–offers a distinct advantage for investigating OBEs over other contemporary laboratory protocols. Virtual reality (VR) paradigms that induce bodily detachment into an external mannequin or avatar (Blanke and Metzinger, 2009; Ehrsonn, 2007) generate an OBE directed toward the *rear* of the mannequin. In contrast, the eye-to-eye setup enables subjects to observe the OBE face-to-face, from a direct, frontal perspective. Consequently, this paradigm more faithfully models clinical cases of autoscopic OBEs, wherein individuals confront their detached self-image face-to-face, as if looking through “non-reflecting mirrors” (Brugger, 2002). Because classic VR protocols relying on synchronous visuo-tactile stimulation (Petkova and Ehrsson, 2008; Lenggenhager et al., 2007) generate posterior-view perceptions, they are more accurately classified as “pseudo-OBEs.” True autoscopic hallucinations require a frontal orientation relative to the out-of-body self (Brugger, 2002; Blondiaux et al., 2021). The eye-to-eye setup provides precisely this frontal viewing condition while eliciting robust apparitional experiences that trigger genuine empathy, emotional resonance, and dissociative identity phenomena.

Beyond its utility in modeling OBEs, the strange-face paradigm provides a valuable framework for examining visual hallucinations along a continuous clinical spectrum–ranging from low-level sensory after-effects (e.g., neon color spreading) to complex, expectation-driven hallucinations (Rogers et al., 2021). Apparitional mirror-gazing experiences have already attracted diagnostic interest for their potential to operationalize early psychotic phenomena (Caputo et al., 2012; Poletti et al., 2026). Specifically, the task offers a standardized laboratory method to elicit mirror-based hallucinations, potentially formalizing the clinical assessment of the classic “mirror sign” in schizophrenia originally described by Abély (1930).

The propensity to experience these phenomena is heavily mediated by individual differences in absorption–the personality trait and cognitive capacity for total immersion in sensory, imaginative, or mental states (Tellegen and Atkinson, 1974; Dalenberg et al., 2022). In the eye-to-eye context, high absorption intensifies sustained focus on the partner’s face, specifically amplifying depersonalization and the felt detachment of the face from the body, i.e., specifically increasing OBEs (Caputo and Daniels, 2026). Continuous fixation under dim lighting may overload the specialized neural architecture dedicated to face processing (Zhao et al., 2018), with high absorption accelerating this sensory adaptation and precipitating the onset of OBEs.

Under typical conditions, exteroceptive input anchors attention firmly to the external environment. In dim eye-to-eye gazing, however, the attenuation of exteroceptive signals prompts a functional shift toward interoceptive bodily channels. While interoception was traditionally restricted to visceral sensing, modern frameworks incorporate skin afferents processing affective touch, temperature, and nociception (Craig, 2003), working in tandem with proprioception to map bodily position and agency (Tuthill and Azim, 2018). Although connections between interoception and dissociation have been noted previously (Woelk and Garfinkel, 2024), prior research has failed to isolate the precise gating role of OBEs revealed here. Our findings suggest that during an OBE, the boundary between the subject’s facial skin schema and that of the partner becomes transiently permeable. This somatic boundary blur allows the subject to physically embody the partner’s bodily feelings and affective states, creating the somatic substrate required to project internal memory prototypes onto the partner’s face.

This cross-body identification–demonstrated by participants recognizing their own face within the partner’s features (facial “fusion” of SFQ item 15)–closely parallels the classic “enfacement illusion” typically induced via synchronous tactile stroking during mutual gaze (Porciello et al., 2018). Furthermore, our data reveal a dynamic dialectical tension between self-other fusion (item 15) and self-other differentiation (item 5), capturing a complex psychological boundary shift that comes from pre-projection out-of-body states. This provides empirical support for our hypothesis of an experiential “enfolding” or somatic blending of facial skin schemas between dyadic partners.

Sustained dyadic gazing over a 10-min period under physical stillness shares procedural features with contemplative and relational mindfulness practices. Similar attentional mechanisms are utilized in *Trataka* (yogic candle-gazing; Swathi et al., 2021) and Mindful Face Gazing, both of which involve social engagement systems to foster interpersonal trust, empathy, and vagal regulation (Ludwig et al., 2025). These practices rely on a steady, soft gaze to calm the autonomic nervous system and cultivate present-moment awareness, often inducing autonomic synchronization of respiratory rhythms between partners (Järvelä et al., 2021; Schussel, 2026). However, whereas clinical and contemplative mindfulness techniques are conducted under normal ambient lighting to promote grounded awareness, our paradigm utilizes low-level illumination specifically to disrupt perceptual constancy, encourage depersonalization, and probe the neurodynamics of altered self-experience (Caputo et al., 2021).

Finally, our findings demonstrate that within the eye-to-eye gazing paradigm, the Self and its associated dissociative identity states manifest as distinct, partially independent phenomenological prototypes. We obtained clear empirical validation for specific prototypes, including the *Adolescent*, the *Ancestor*, and the *Child*. The eye-to-eye laboratory setup thus acts as a specialized contemplative engine, facilitating the simultaneous emergence of the immediate “experiential Self” and the temporally extended “narrative Self” (Gibson, 2024). Dyadic OBEs appear to arise from a shared interoceptive state (Woelk and Garfinkel, 2024) driven by implicit facial mimicry (Carr and Winkielman, 2014; Hess and Fischer, 2022). Once an OBE occurs and a projection of an “experiential” prototype onto the partner’s face arises, the participant’s “narrative Self” constructs a coherent retrospective attribution to integrate and explain the unsettling apparitional experience.

In summary, why must a psychological projection be preceded by an OBE? Somatosensorial and phenomenological considerations indicate that the participant’s felt bodily boundary–their facial “skin schema”–must enlarge to “enfold” the partner’s physical presence by somatic blending of facial skin schemas. This temporary suspension of self-other boundaries creates the necessary somatic matrix to bind subjective mental representations and prototypes onto an external face.

### Archetypes

The diverse prototypes elicited in this paradigm reflect not only specific autobiographical memories but also broader archetypal structures (Caputo, 2014). Viewed through the lens of Jungian analytical psychology, a direct conceptual mapping emerges between the projected figures of the *Ancestor*, *Adolescent*, *Child*, and *Monster*, and Jung’s foundational archetypes: the Wise Master (*Philemon/Baucis*), the Syzygy (*Anima/Animus*), the Divine Child (*Puer Aeternus*), and the Shadow, respectively (Jung, 1971, 1979, 2009). Path Analyses demonstrate that “negative” archetypes, such as the Shadow, are projected rapidly onto the partner’s face as dark, threatening figures. Conversely, “positive” archetypal projections–the Wise Master, Syzygy, and Divine Child–are more elusive, requiring specific depersonalization conditions to emerge and remain sustained.

Cross-cutting qualities of spirituality (SFQ item 14) and heroism (item 6) are shared across both Ancestor and Child apparitional experiences. These overlapping traits reflect a recognized archetypal continuum linking the two figures (Hillman, 2005). Specifically, the perceived visual “enlightenment” of the partner’s face (item 14)–phenomenologically characterized as an epiphany of spiritual radiance–can be understood as “the shining,” a visual manifestation of this shared transcendent quality.

As illustrated in Figure 6, the three primary positive archetypes load onto partially distinct statistical factors. This structural independence is most pronounced between the Wise Master and the Syzygy. However, the Divine Child bridges these domains by retaining shared loadings on enlightenment and wisdom traits with the Wise Master.

A classic *conjunctio oppositorum* (union of opposites) is evident in the projection of the Adolescent (item 9) onto the partner’s face: feelings of identity fusion (item 15) co-occur with feelings of estrangement (item 5), both converging on the archetype of the Syzygy. The Syzygy (*Anima/Animus*) captures the immediacy and vitality of the present moment. In dyadic interactions, adolescent love is uniquely characterized by an intense impulse for boundary dissolution alongside an opposing drive for self-differentiation. This dynamic aligns directly with Jung’s (1979) formulation of the *Anima* and *Animus* as an archetypal syzygy of contrasexual opposites–representing the inner feminine in the male psyche and the inner masculine in the female psyche. Consequently, the dyadic eye-to-eye paradigm is uniquely suited to constellate the *Anima/Animus* archetype within the participant dyad. This archetype may also be designated as “Perennial Youth,” as it is intrinsically bound to psychological renewal, capturing both the hopeful vulnerability of relational intimacy and the painful sting of betrayal.

Crucially, our quantitative results demonstrate that specific archetypes organize into compensatory paired structures. Beyond the *Anima/Animus* pair, the *Senex/Puer* polarity emerged statistically from the Ancestor/Child prototype pair via the shared intermingling of heroic and spiritual features. The empirical confirmation of these compensatory polarities strongly supports Jung’s (1971) original structural model of the psyche. While this compensatory organization is facilitated by the dyadic setup, it represents an intrinsic feature of human cognitive architecture, historically reflected in Gnostic dualism (Jung, 1979).

A fundamental tension emerging from these empirical data is the polarity between spirituality and materiality. This contrast reflects the dialectic between positive and negative projections as conceptualized in analytical psychology (Jung, 1971). Rather than indicating simple Gnostic dualism (Jung, 1979), this polarization captures the psychological tension between the Self and the Shadow of the Self–specifically, the latter representing the externalized “materialization” of one’s unintegrated psychological darkness (i.e., the Shadow) and the perversion of spiritual fulfillment.

Additional non-dualistic archetypal prototypes also emerged from SFQ endorsements: specifically, the *Hermaphroditus* and the *Animal*. The *Hermaphroditus* archetype is anchored by SFQ item 10 (“androgyne”), which clusters with item 13 (“another race”) and items from the adolescent cluster (e.g., item 9; see Figures 8A, 9). Path Analysis (Figure 8B) reveals the characteristic profile of positive archetypes: inhibition by derealization (DR) combined with mediation by OBEs. For Jung (1971), the *Hermaphroditus* served as the primary visual symbol of the *conjunctio oppositorum* between *Anima* and *Animus*. The empirical intersection between the *Hermaphroditus* and Adolescent prototypes provides direct quantitative support for our *Anima/Animus* framework (Figure 9).

**FIGURE 8:**
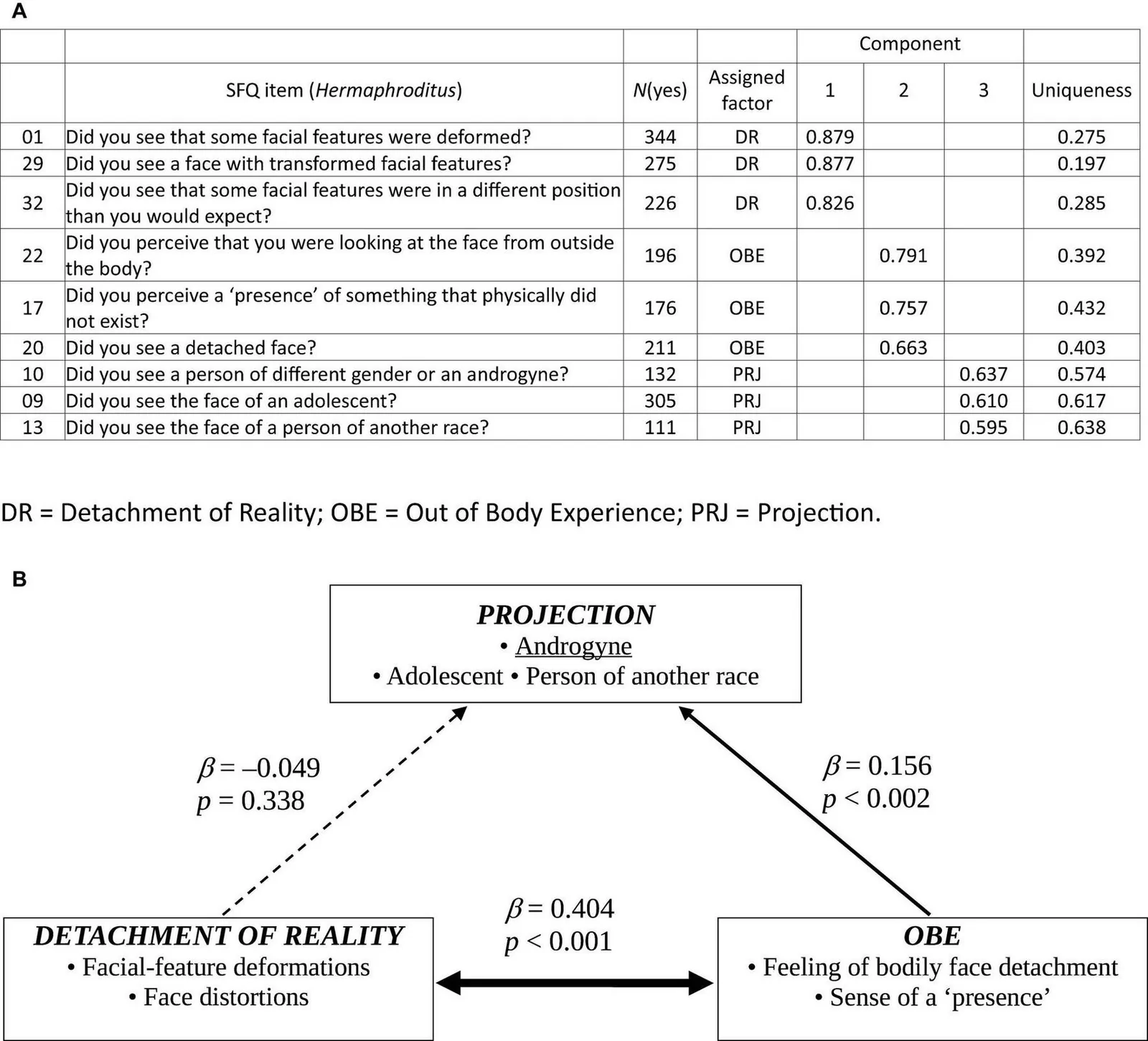
**(A)** Results of a Principal Component Analysis. A factor analysis yielded two fixed factors (DR and OBE) and a third factor (Projection). This third factor centered on the apparitional experience of an “Androgyne” (item 10) projected onto the face of the other participant in the dyad. This prototype clustered with other items, characterizing the androgyne as an adolescent (item 9) or a person of another race (item 13). The resulting model accounted for 58% of the total explained variance. **(B)** Path Analysis for the “*Hermaphroditus*” prototype. Apparitional projections of alternative personalities–such as latent dissociative identities of an androgyne, an adolescent, or a person of another race–become “embodied” within the other’s face in the dyad.

**FIGURE 9:**
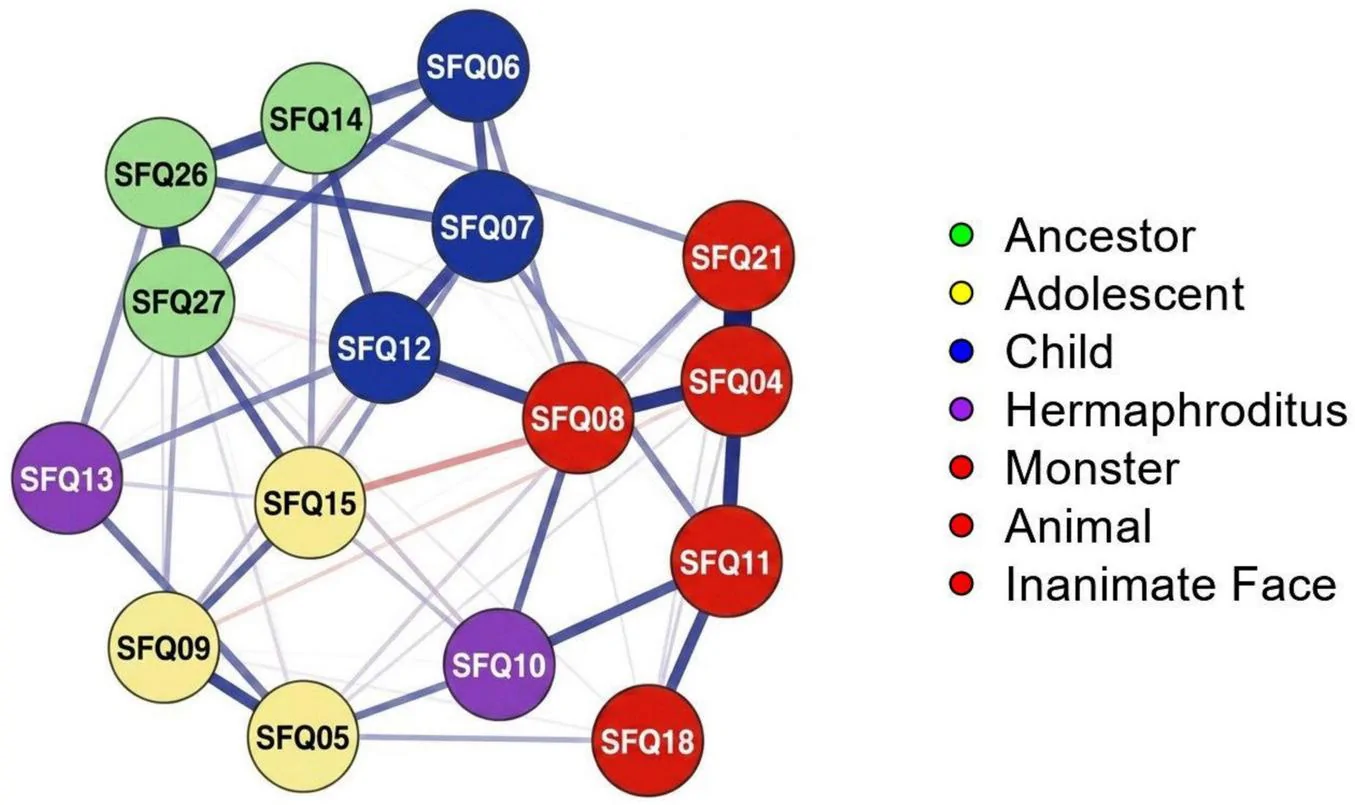
Network Analysis of the seven prototypes that have emerged from SFQ in the present study. “Positive” projections occupy the left side of the network, while “negative” projections–all shown in red–cluster on the right. At the top sits the Shining Heroic Child (items 14, 6, and 7), and at the bottom is the deadly Inanimate Face (item 18). Notably, this network highlights several key features: (1) a “regression” from the Alien (item 21) to the Monster (item 4) and the Animal (item 11), moving downward the deadly Inanimate Face (item 18); (2) a striking opposition between adolescent fusion (item 15) and old person’s degeneration (item 8); (3) a progressive growth from the Adolescent (item 9) toward the enlightened Ancestor (item 26) and Child (item 7); (4) an Androgyne (item 10) gravitates toward the *Anima*/*Animus* zone (centered on the prototype of an Adolescent, item 9), and is suspended in equilibrium between the Animal (item 11) and the Stranger (item 5); Another Race (item 13) is also attracted to this cluster. Note the lack of a direct connection between the spiritual face Shining (item 14) and the *Hermaphroditus* (items 10 and 13), and only a moderate link between the spiritual face Shining (item 14) and item 15 (lovers’ fusion) of the *Anima*/*Animus* syzygy (items 9, 5, and 15). Consequently, the *Anima*/*Animus* syzygy, which we have termed Perennial Youth, might also be called Perpetual Deceit–the very source of illusions and delusions (Jung, 1979).

The *Animal* archetype is anchored by SFQ item 11 (“animal”) and clusters with item 8 (“old person”), channeling the negative, material dimensions of old people (Figure 10A). Path Analysis (Figure 10B) reveals an excitatory link with derealization (DR) alongside a diminished reliance on OBEs during the emergence of this negative archetype. In this sense, the *Animal* functions similarly to the Shadow, representing uncultivated instinctual drives such as aggression, territoriality, and raw sexuality. Although integrating the *Animal* archetype allows the ego to reclaim vital psychic energy (Hillman and McLean, 1997), the initial encounter is often perceived as a regression or degradation of humanity–explaining its empirical clustering with aging (“old person”).

**FIGURE 10:**
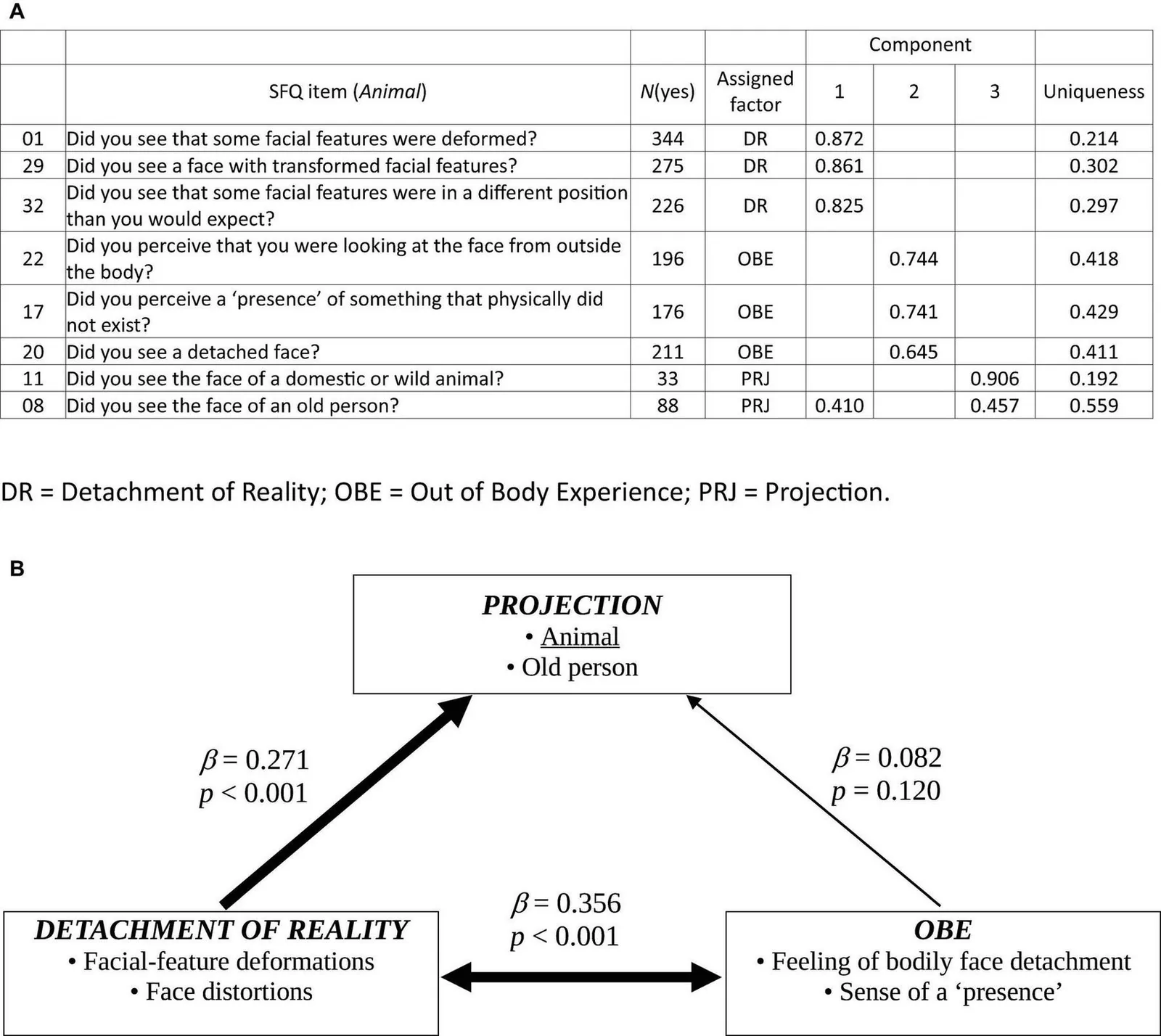
**(A)** Results of a Principal Component Analysis. A factor analysis yielded two fixed factors (DR and OBE) and a third factor (Projection). This third factor centered on the apparitional experience of an “Animal” (item 11) projected onto the face of the other participant in the dyad. This prototype clustered with another item, characterizing the animal as an old person (item 8). The resulting model accounted for 65% of the total explained variance. **(B)** Path Analysis for the “*Animal*” prototype. Apparitional projections of alternative personalities–such as latent dissociative identities of an animal or an old person–become “embodied” within the other’s face in the dyad. The apparitional Animal is a “negative” projection, because much like Monsters, it has a positive link leading from detachment of reality (DR) to projection.

Finally, the *Lapis*–representing the inanimate, inorganic dimension of the facial substrate–is captured by item 18 of the SFQ (Figure 11A). Although no other items cluster with it, a substantial majority of participants (322 out of 398) endorsed this item, establishing it as an independent single-item prototype reflecting the impermeable indifference of the alchemical *Lapis*. Path Analysis confirms that the *Lapis* conforms to the characteristic statistical profile of a negative archetype (Figure 11B). Perceiving an ‘immobile face’ (item 18) is akin to gazing at an inanimate statue–another symbol of the Lapis (Jung, 1971, chap. 5.2)–and when this inanimate statue would be enfolded by light of the Sol at noon (i.e., spiritual Shining, item 14), the association reinforces its character of immutable perfection and insensible divinity–in fact, the hardest conjunctio oppositorum (given the opposing distance of the two prototypes: see Figure 9).

**FIGURE 11:**
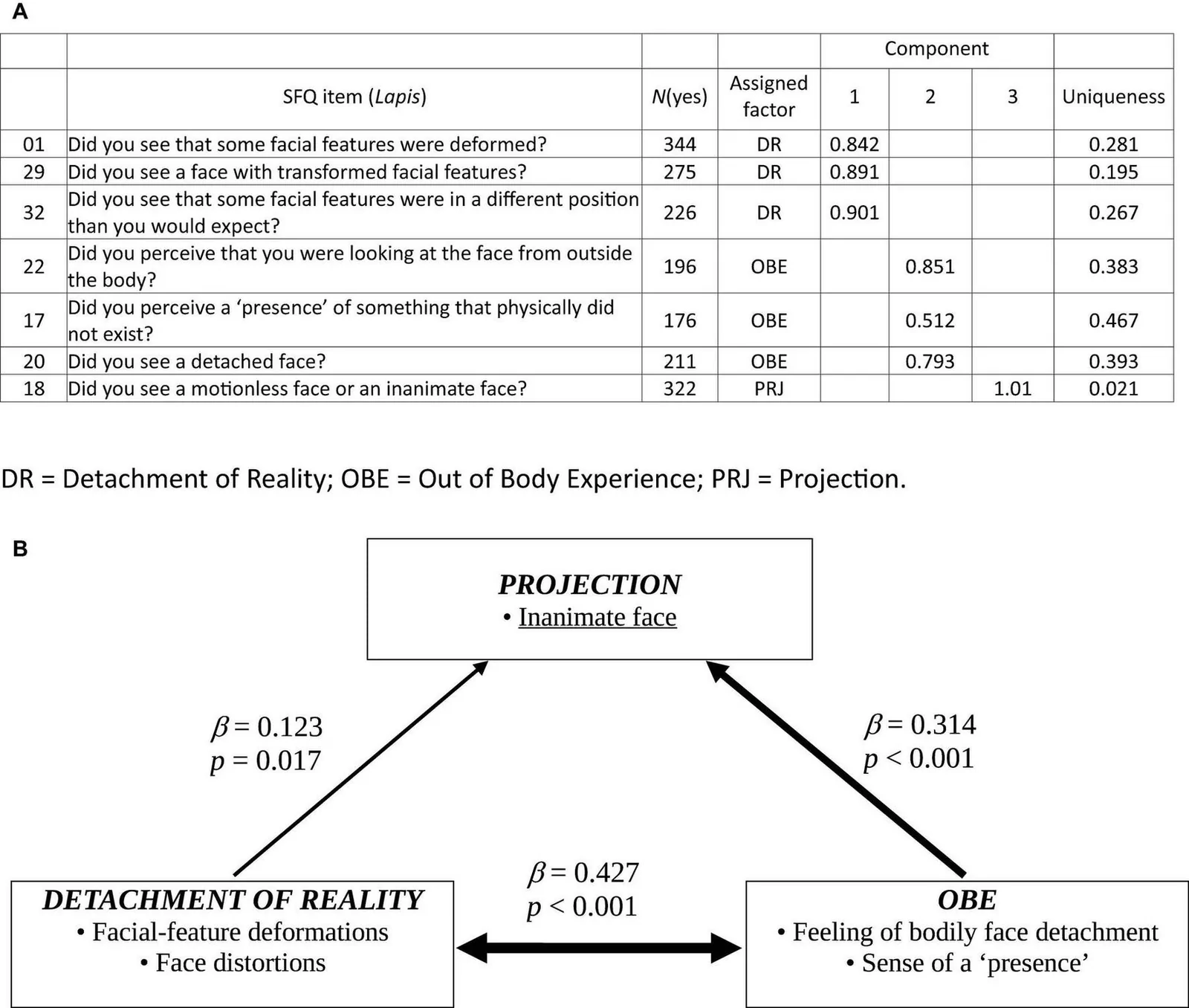
**(A)** Results of a Principal Component Analysis. A factor analysis yielded two fixed factors (DR and OBE) and a third factor (Projection). This third factor centered on the apparitional experience of an “Inanimate face” (item 18) projected onto the face of the other participant in the dyad. This prototype clustered with no other SFQ item. The resulting model accounted for 71% of the total explained variance. **(B)** Path Analysis for the “*Lapis*” prototype. The apparitional projection of an “Inanimate face” becomes “embodied” within the other’s face in the dyad. The apparitional inanimate face is a “negative” projection, because like Monsters and Animals it has a positive link leading from detachment of reality (DR) to projection.

A striking parallel exists between the perception of the *Lapis* (i.e., the “inanimate face” of item 18) and the clinical presentation of Cotard’s syndrome in neuropsychiatry (Debruyne et al., 2009). Cotard’s syndrome is a severe condition characterized by nihilistic delusions, wherein patients maintain that they are dead, putrefying, or devoid of an internal soul (i.e., the extremization of materialism). Neurobiologically, dysfunction within the temporoparietal junction (TPJ) and insular cortex impairs the integration of interoceptive bodily signals with exteroceptive inputs, leading to the false inference of non-existence. Disruptions in connectivity between the prefrontal cortex (governing reality testing) and limbic structures embed these nihilistic beliefs. Because the projection of inanimate, deathlike inanimate faces occurs reliably during low-illumination gazing, we hypothesize a shared, transient neurocomputational mechanism. Specifically, Figure 11B points to a temporary out-of-body state–triggered by hyper-excitation within the insula and TPJ–co-occurring with a detachment from reality driven by disinhibition in prefrontal-temporal (ventral “what” stream) and prefrontal-parietal (dorsal “where” stream) feedback circuits. In psychiatric populations, this neurofunctional disruption may explain the clinical fascination with mirrors (“mirror sign”; Abély, 1930) and the unexplained experience of multiple faces filling the mirror space during mirror-gazing tasks in some patients diagnosed with schizophrenia (Caputo et al., 2012).

Non-dualistic archetypes–such as the *Hermaphroditus*, *Animal*, *Rotundum*, or the inorganic deadly *Lapis*–may emerge more readily within mirror-gazing setup. In contrast, the dyadic eye-to-eye paradigm is uniquely optimized for constellating interpersonal, relational archetypes, most notably the *Anima/Animus* syzygy.

In conclusion, these empirical findings refine and extend earlier theoretical models of archetypal imagery (Caputo, 2014). By demonstrating a fundamental distinction between positive and negative projections, this research highlights the essential role of OBEs in facilitating positive archetypal projections. Because contemplative and spiritual traditions historically emphasize physical detachment and somatic transcendence (states functionally analogous to pre-projective OBEs), our findings clarify how these practices facilitate the projection of transcendent archetypes–ultimately revealing the “shining,” transfigured faces of spiritual experience of the Others.

### Limitations

A first methodological limitation concerns the absence of strict foveal gaze monitoring between dyad members within the laboratory setup. Precise fixation control could be readily implemented in a mirror-gazing paradigm (Caputo, 2023) by placing a fixation target (e.g., an aperture a few millimeters in diameter) directly between the eyes of the subject’s reflection, with a video camera mounted behind it for continuous gaze tracking. Such a modification would likely increase the frequency and salience of strange-face illusions while enhancing OBEs through heightened attentional “absorption” in the fixation point. In essence, narrowing attentional focus on an explicit fixation target may constrain “absorption” of exteroceptive processing, thereby uncoupling exteroceptive constraints and amplifying interoception-driven OBEs (Caputo and Daniels, 2026).

A second limitation involves the random pairing procedure utilized to form participant dyads. Because biological sex and gender identity were not controlled during dyadic assignment, we were unable to examine subtle individual differences or relational dynamics associated with these variables. Similar constraints apply to other demographic factors, including race, ethnicity, socioeconomic status, religious background, and broader cultural context. Given the procedural complexity required to fully counterbalance these dyadic variables, future research might investigate these individual differences more systematically using a standardized mirror-gazing paradigm (Caputo, 2023).

A third limitation relates to sample composition, which consisted exclusively of first-year undergraduate students. This reliance on a relatively homogeneous non-clinical population limits the generalizability of our findings across broader age cohorts, as well as clinical populations presenting with neurological, neurodegenerative, or psychiatric conditions. Future studies should address this constraint by evaluating strange-face illusions within more demographically diverse and clinically varied samples.

Finally, the present study relied exclusively on psychometric self-report measures, omitting objective physiological and electrophysiological markers. Given the exploratory nature of this investigation, our primary objective was to map and characterize the psychological and phenomenological architecture of strange-face illusions. Future research can build upon these psychometric foundations by incorporating objective autonomic measures and electroencephalographic (EEG) recordings to elucidate the neural correlates of dyadic dissociative states.

## Conclusion

The dyadic eye-to-eye laboratory setup reliably induces face-specific illusions characterized by three distinct psychometric dimensions: the detachment of external reality (derealization), out-of-body experiences (depersonalization/OBE), and the apparitional projection of novel identities onto the partner’s facial substrate. Within this paradigm, participants experience a frontal autoscopic OBE that closely mirrors clinical hallucinations observed in neuropsychiatric populations. Notably, Path Analyses demonstrates that these OBEs serve as a pivotal mechanism driving the projection of alter-identities onto the partner within the dyad.

### Network architecture and archetypal topology

These results indicate that dyadic eye-to-eye gazing allows complex, multifaceted projections to flourish. These projective identities exhibit partial statistical independence from one another. From a cognitive perspective, these prototypes can be conceptualized as a network capturing the diverse apparitional experiences elicited during the task (Figure 9). “Positive” projections occupy the left side of the network topology, whereas “negative” projections–rendered in red in Figure 9–cluster on the right. This topological structure reflects the intrinsic nature of the symbols underlying strange-face illusions. As Jung (1961) observed, a psychological symbol cannot be reduced to a single definition; rather, it opens onto a vast array of fluid, interconnected variations.

Crucially, the Network Analysis of Figure 9 clarifies the absence of a direct statistical connection between the spiritual “Shining” prototype (item 14) and the *Hermaphroditus* (items 10 and 13), as well as the minimal linkage between the “Shining” and romantic “fusion” (item 15) within the *Anima/Animus* syzygy (items 5, 9, and 15). Jung (1979) originally conceptualized the *Anima/Animus* syzygy as a primary source of human deception–analogous to the concept of *Maya* in Eastern philosophical traditions, representing the cosmic force that renders the exteroceptive world seemingly solid despite its impermanent, illusory nature. While post-Jungian theorists such as Hillman (1985) reframed the *Anima* as an exclusively positive archetype, our network topology aligns directly with Jung’s original formulation by demonstrating that the *Anima/Animus* cluster remains distinct from pure spiritual transcendence.

Several anthropological insights emerge from the structural configuration of this archetypal network (Figure 9). When the Adolescent (*Anima/Animus*) and Androgyne (*Hermaphroditus*) items are combined into a single cluster representing *Love* (including the “fusion” of races, item 13), and the three negative projections (*Monster*, *Animal*, and *Lapis*) are unified as the negative *Shadow*, a distinct quadripartite factor structure emerges. This configuration yields three elements on the positive continuum–the *Wise Master*, the *Divine Child*, and *Love*–and one on the negative continuum: the *Shadow*. This structure empirically supports Jung’s (1969) hypothesis that archetypal structures and their cultural expressions (such as mandalas) exhibit a fundamental quaternity (3 + 1). In this model, three components represent conscious, differentiated psychic functions (*Wise Master*, *Divine Child*, *Love*), while the fourth remains dark, unconscious, and unintegrated (*Shadow*, encompassing the *Monster*, *Animal*, and *Lapis*). Across cultural cosmologies, kinship systems, and spatial orientations, the number four consistently organizes human perception of reality, symbolizing structural completeness and balanced polarities (Lévi-Strauss, 1969; Eliade, 1971). This structural quaternity manifests universally across human cultures as well as during strange-face illusions–as if the human face itself projects its own “effigy” onto the cosmos like a mirror reflection (Dante, *Paradiso*, Canto XXXIII, line 131).

### Individual differences and somatosensory grounding

Endorsement frequencies (*N*(yes)) across items reveal substantial inter-individual variability, likely reflecting participants’ developmental positioning along a continuum of materiality versus spiritual transcendence. While the vast majority of participants endorsed the *Lapis*–the prototype furthest removed from spiritual transcendence and anchored in deadly inorganic materiality–considerably fewer endorsed the *Child* or *Ancestor* prototypes, and fewer still identified with the *Hero/Heroine* or *Spiritual* items. This marked disparity points to significant individual differences in spiritual transcendence as a fundamental personality trait (Piedmont, 1999), standing in sharp contrast to materialisms.

Methodologically, eye-to-eye gazing demonstrates remarkable efficacy in eliciting deep empathy and affective resonance, offering a robust paradigm to explore how the “experiential” and “narrative” selves construct identity (Gibson, 2024). The somatosensory connection established during dyadic gazing is sufficiently potent that participants frequently report a felt blurring of physical boundaries–as though their facial skin schema were physically “embracing” their partner’s, enabling them to directly embody the other’s affective states. Remaining stationary while maintaining a soft, continuous gaze parallels contemplative practices such as *Trataka* and Mindful Face Gazing. However, while traditional mindfulness techniques are typically conducted under ambient lighting to cultivate interpersonal trust and autonomic regulation, adapting this protocol to low-light conditions and neutral facial expressions transforms it into a specialized tool for probing altered states of consciousness and integrating unconscious mental content.

Somatic markers–most notably respiratory rhythm–serve as a critical bridge uniting interoceptive, proprioceptive, and exteroceptive sensory channels (including visual, acoustic, and tactile perception of warm breath movements against the skin, as well as gently “caressing the skin” by breathing). This multisensory feedback loop grounds the conscious perception of a living presence, fundamentally distinguishing the dyadic partner from a static photograph. Both traditional meditative disciplines and low-light gazing rely on these somatic facets of respiration to anchor present-moment awareness and facilitate implicit psychological integration.

### Psychometric challenges, clinical parallelism, and transformation

In summary, the projective identities elicited in this paradigm divide cleanly into positive and negative archetypal categories that map directly onto Jungian analytical psychology:

1. Positive Projections: Archetypes such as the *Wise Master* (perceived as an Ancestor), *Perennial Youth* (an Adolescent), the *Divine Child*, and the *Hermaphroditus* emerge from autobiographical memory or latent face schemas. However, these positive projections are notoriously difficult to evoke and require sustained somatosensory disembodiment (OBE) to maintain.
2. Negative Projections: Archetypes such as the *Monster*, *Animal*, and deadly *Lapis* emerge rapidly as sudden, intrusive perceptual bursts. For these negative projections, high levels of derealization amplify the illusion, fostering a heightened delusional conviction that the monstrous apparition is more real than the partner’s actual face.

Previous Path Analyses can be summarized. Figure 12 depicts separate Path Analyses for “positive” *versus* “negative” projections, where average *z*-scores were used to determine the mean strength of the links. It should be noted that it is important to keep separated the two types of projections.

**FIGURE 12:**
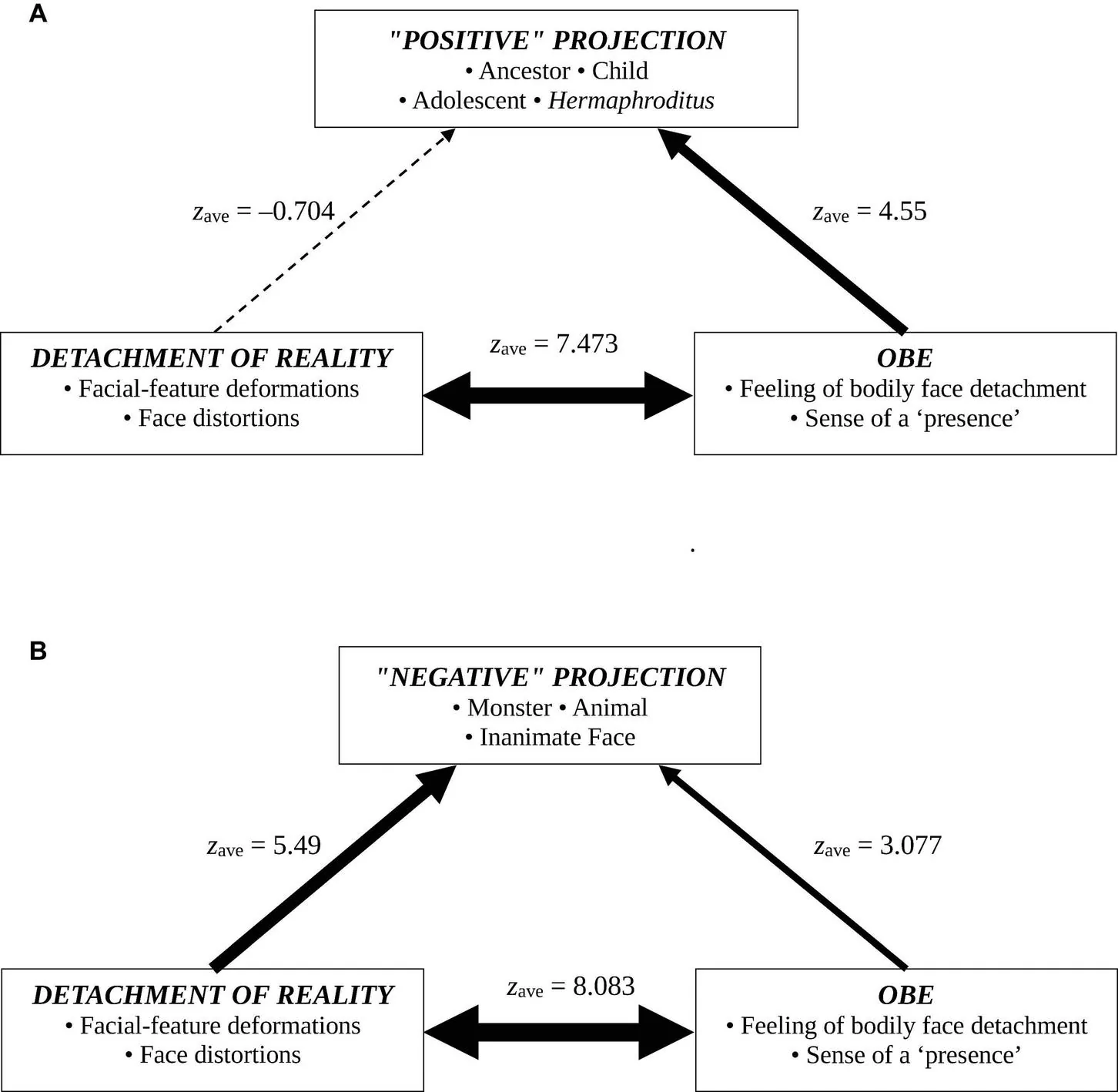
**(A)** Averaged Path Analyses of “positive” projections. **(B)**. Averaged Path Analyses of “negative” projections.

From a psychometric perspective, the extraction of seven distinct, partially independent prototypes across the SFQ items presents a challenge for establishing a single, unidimensional scale for face projections. Developing a comprehensive, dedicated metric for this third dissociative factor–to complement the standardized derealization and depersonalization subscales (Caputo and Daniels, 2026)–remains an important objective for future research. Nevertheless, these findings demonstrate that the projections induced by low-light dyadic gazing involve multiple, polymorphic identity states that characterize the landscape of collective dissociative identity.

In clinical contexts, these same mechanisms may pivot into severe psychopathology, such as dissociative identity disorder (DID). Trauma survivors with DID routinely experience sudden autoscopic OBEs when confronted with threat cues (Mudgal et al., 2021; Bravo et al., 2023), accompanied by derealization of the perpetrator and the abrupt projection or emergence of an alternate identity state (“alter”). In our data, additional SFQ items measuring time distortion and loss of agency support the hypothesis that non-conscious facial mimicry (Hess and Fischer, 2022) and non-conscious mirroring (Carr and Winkielman, 2014) underpin dyadic dissociative states.

Comparing the Path Analyses of positive prototypes (*Ancestor*, *Adolescent*, *Child*, *Hermaphroditus*) against negative ones (*Monster*, *Animal*, *Lapis*) reveals a clear regulatory trade-off in how derealization (DR) and out-of-body states (OBE) trigger projections. While the path from OBE to Projection is consistently positive across all figures–confirming the mandatory role of somatic disembodiment in anchoring projective content–derealization operates differentially based on valence. Specifically, DR exhibits an inhibitory (negative) link to positive projections (Figure 12A), but an excitatory (positive) link to negative ones (Figure 12B). Consequently, facilitating a transition from a negative projection toward a positive one requires down-regulating derealization–which may be achieved by deepening attentional “absorption” in the partner’s face (Caputo and Daniels, 2026) and regulating autonomic breathing rhythms to stabilize the out-of-body state.

Alternatively, Figure 9 outlines a developmental roadmap for dyadic open-eye meditation, tracing a progression through key archetypal transformations. The journey begins by confronting the *Monster* (item 4)–a stage that requires relinquishing projections of the *Alien* (item 21), raw instinctual threats of the *Animal* (item 11), and the uncanny coldness of the inanimate *Lapis* (item 18). From there, the individual moves through the *nigredo* of decay represented by an old person (item 8), sheds the idealizations of “fusional” *Love* (item 15), and transcends the charming lure of the *Androgyne* (item 10). Progressing further, the participant confronts representations of *Parents and Relatives* (item 12), ultimately reaching a transformative threshold: giving birth to the heroic *Divine Child* (item 7) or ascending toward the spiritual “Shining” (item 14) of the *Ancestors*. In the language of classical alchemy, this psychological progression models the *Magnum Opus*–the gradual transmutation of dense psychic lead into spiritual gold.

## Data Availability

The original contributions presented in this study are included in this article/supplementary material, further inquiries can be directed to the corresponding author.
